# Quantification of the effect of environmental changes on the brownification of Lake Kukkia in southern Finland

**DOI:** 10.1007/s13280-023-01911-7

**Published:** 2023-09-21

**Authors:** Katri Rankinen, Virpi Junttila, Martyn Futter, José Enrique Cano Bernal, Daniel Butterfield, Maria Holmberg

**Affiliations:** 1 Finnish Environment Institute, Latokartanonkaari 11, 00790 Helsinki, Finland; 2 Swedish University of Agricultural Sciences, P.O. Box 7070, 750 07 Uppsala, Sweden; 3 Enmosys, Vermont, USA

**Keywords:** Atmospheric deposition, Brownification, Climate change, Land-use change, Process-based models

## Abstract

The browning of surface waters due to the increased terrestrial loading of dissolved organic carbon is observed across the northern hemisphere. Brownification is often explained by changes in large-scale anthropogenic pressures (including acidification, and climate and land-use changes). We quantified the effect of environmental changes on the brownification of an important lake for birds, Kukkia in southern Finland. We studied the past trends of organic carbon loading from catchments based on observations taken since the 1990s. We created hindcasting scenarios for deposition, climate and land-use change in order to simulate their quantitative effect on brownification by using process-based models. Changes in forest cuttings were shown to be the primary reason for the brownification. According to the simulations, a decrease in deposition has resulted in a slightly lower leaching of total organic carbon (TOC). In addition, runoff and TOC leaching from terrestrial areas to the lake was smaller than it would have been without the observed increasing trend in temperature by 2 °C in 25 years.

**Supplementary Information:**

The online version contains supplementary material available at 10.1007/s13280-023-01911-7.

## Introduction

The browning of surface waters due to the increased terrestrial loading of dissolved organic carbon (DOC) is observed across the northern hemisphere. This increase has been widely studied (see, e.g. Härkönen et al. 2023) as it may form a considerable part of the areal carbon balance (de Wit et al. 2015). Thus, it has raised a debate about the factors controlling the export of DOC from catchments (Roulet and Moore 2006). Brownification is often explained by changes in large-scale anthropogenic pressures and ecosystem functioning, including acidification, climate change and land-use changes. There have been considerable changes in these environmental pressures during recent decades.

Atmospheric sulphur (SO_4_) deposition has decreased considerably since the 1990s due to a successful air protection policy. A decrease in SO_4_ deposition is assumed to increase organic soil matter solubility (de Wit et al. 2007; Monteith et al. 2007). Thus, increased DOC loading is assumed to result from a process of recovery in terrestrial ecosystems. The observed increases in DOC have also been linked to climate change. Suggested climatic drivers of the upward trends have included temperature (Freeman et al. 2001; Evans et al. 2006), soil moisture impacts on decomposition processes (Worrall et al. 2006) and variation in the timing and intensity of precipitation and snowmelt (Hongve et al. 2004; Erlandsson et al. 2008). Land-use changes and forestry measures (Mattsson et al. 2005; Nieminen et al. 2020; Estlander et al. 2021; Nieminen et al. 2021; Škerlep 2021; Williamson et al. 2021; Cano Bernal et al. 2022) have recently been observed to form one reason for increased concentrations of DOC and the transport of DOC. According to Škerlep (2021), increases in coniferous forest cover is also an important and overlooked factor behind browning. In particular, spruce forest promotes the accumulation of organic soils and the mobilisation of dissolved organic matter (DOM). The effect is seen also at small catchment level in undisturbed area (Mattson et al. 2002).

Transport processes are usually dominated by hydrology, but decay processes in the terrestrial environment may depend on other controlling factors (see, e.g. Wen et al. 2019). Combinations of these factors can explain many of the observed trends (Erlandsson et al. 2008; Lee et al. 2020), and their order may change along with the time or season (Škerlep et al. 2020; de Wit et al. 2021). The effects of brownification extend to ecosystem services, like water purification, but also to freshwater productivity (Kritzberg et al. 2020) through limiting light penetration (Eloranta 1978) and creating more stable thermal stratification. Thus, changes in DOC concentration and water colour have physical, chemical and biological implications for lake ecosystems and biodiversity (Jones 1992; Arzel et al. 2020), though our understanding of their ecological consequences in different freshwater habitats and regions is still limited (Blanchet et al. 2022).

Process-based biogeochemical models of DOC are tools with which to understand and predict the complex dynamics of organic carbon in soils and surface waters. Landscape-scale models that integrate the contributions of different land-cover and land-use types are required for modelling the fluxes of DOC in aquatic environments. Such models should incorporate the effects of different land-cover types on DOC generation, simulate the effects of atmospheric deposition and climate on DOC fluxes, simulate the production and consumption of organic carbon in soil, and represent the biological and chemical processes that consume DOC in surface waters. Typically, they are also used to make predictions for the future under changing environmental conditions (Futter et al. 2009).

In this study our main aim was to quantify the effect of past environmental changes on the brownification of an important lake for birds, Lake Kukkia, located in a boreal forested catchment in southern Finland. Firstly, we analysed long-term observed data on water quality, land use, atmospheric deposition and climate in order to look for possible trends. Secondly, we used Dredge analysis to create a conceptualisation for physically based modelling as it makes an automatic statistical model selection from the data available. We chained physically based models in order to fill gaps in water quality observations and to study the total organic carbon (TOC) loading from catchments. We calibrated two physical eco-hydrological catchment models—the Precipitation, Evapotranspiration and Runoff Simulator for Solute Transport (PERSiST) and the integrated nutrients in catchments (INCA) model (Futter et al. 2007, 2014)—to three small, well-studied catchments at the Lammi long-term ecological research (LTER; Home—LTER at lternet.edu) area and transferred these calibrations to the neighbouring Lake Kukkia catchment. The long-term brownification process of the lake was then studied with the process-based multi-year simulation model for lake thermo- and phytoplankton dynamics (the MyLake model; Saloranta and Andersen 2007). Thirdly, we created hindcasting scenarios for atmospheric deposition, climate change and land-use change in order to simulate their quantitative effect on the brownification of Lake Kukkia.

## Materials and methods

### Area description

**Fig. 1:**
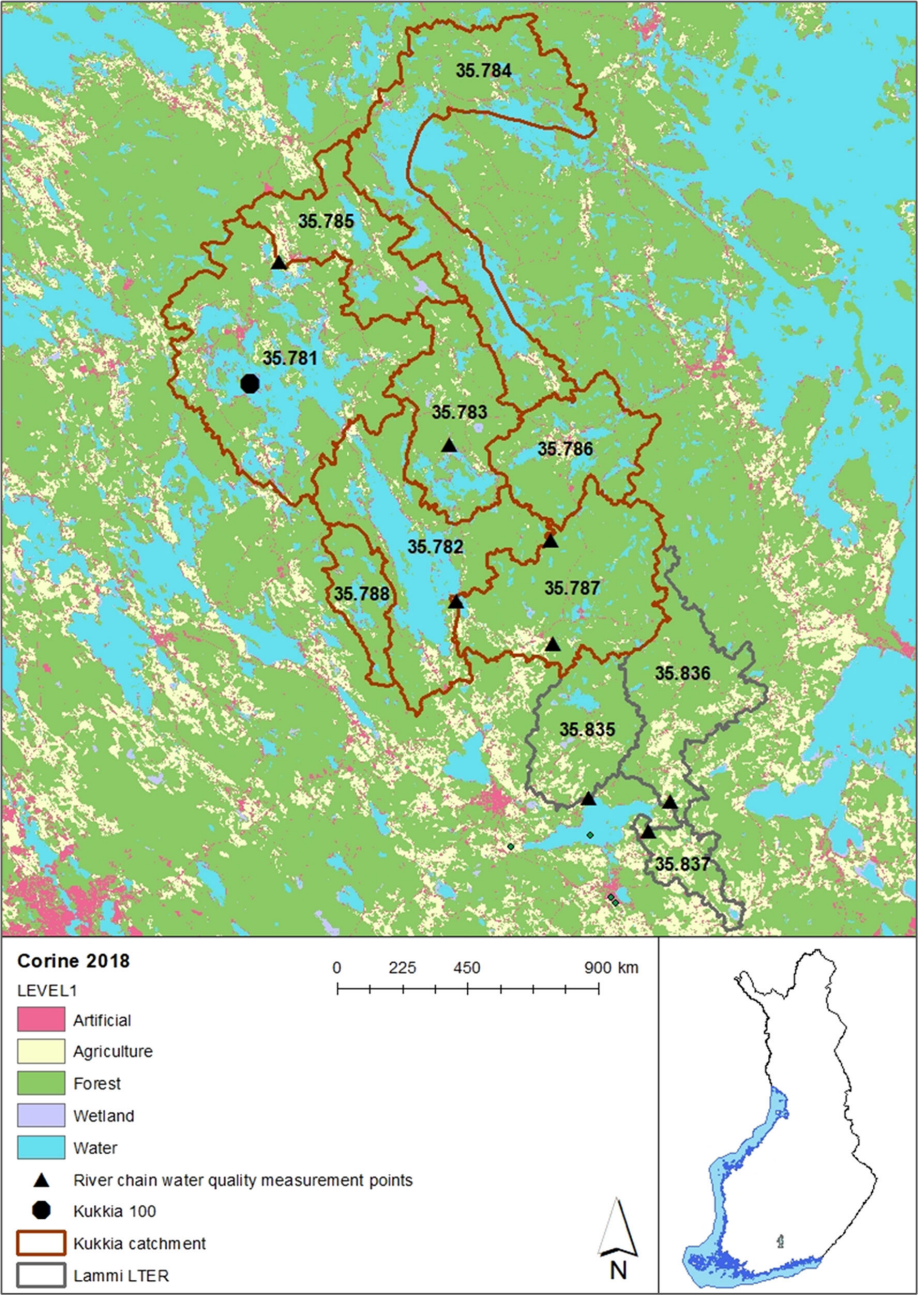
The location of Lake Kukkia and its sub-catchments, and the sub-catchments in Lammi LTER area in southern Finland

As noted, Kukkia lake is situated in southern Finland (ETRMS-TM35FIN 386941, 6790001). The catchment area of the lake (867.58 km^2^) lies in the southern boreal vegetation zone (see Fig. 1). During winter, precipitation typically falls as snow, and the soil is frozen. The annual mean temperature is 4.0 °C and the annual mean runoff is 234 mm (1977–2000; Finnish Meteorological Institute).

The Kukkia lake complex consists of separate, isolated water bodies connected with narrow straits. The total surface water area is 45.75 km^2^ and the shoreline length is 290 km. The mean water depth is 5.7 m, and the deepest point is 34 m (Mäkirinta 1978). The lake complex is mainly oligotrophic with some mesotrophic and eutrophic areas. The main water bodies of Kukkia belong to the Natura 2000 network based on the Habitats Directive (Ministry of the Environment 1999) and to the network of Important Bird Areas (IBAs) in Europe.

The catchment area is approximately 15% water areas and 9% agricultural and built-up areas (see Table 1). The rest of the area is forested, mainly managed forests used for forestry. The main human influence comes through forestry and agriculture. The agriculture there is mainly cereal cultivation. In recent years, the forest cutting area has increased in the catchments. The logging is mainly traditional clear cutting combined with regeneration measures (like mounding) and furrowing and ditch network maintenance treatments in peatland forests.

**Table 1:** Land use in sub-catchments as a percentage of the catchment area, according to CORINE land use

	Kukkia sub-catchments								LTER area sub-catchments		
	35.781	35.782	35.783	35.784	35.785	35.786	35.787	35.788	35.835	35.836	35.837
Urban	4.4%	3.1%	2.7%	3.0%	3.2%	3.3%	1.5%	2.6%	12.7%	14.7%	35.4%
Agriculture	6.2%	4.3%	3.5%	2.2%	8.3%	3.6%	4.6%	1.8%	6.8%	5.6%	7.6%
Forests on mineral soil	52.7%	50.6%	62.5%	54.6%	58.7%	58.5%	68.4%	64.5%	35.3%	36.7%	23.2%
Forests on organic soil	2.9%	5.2%	6.9%	5.6%	8.5%	10.1%	12.0%	5.5%	28.0%	31.9%	21.3%
Forest cutting area	8.1%	7.5%	12.1%	9.5%	12.4%	12.3%	8.0%	12.0%	11.7%	10.1%	10.6%
Open pristine mires	1.1%	0.5%	1.2%	0.2%	1.5%	0.4%	0.6%	0.1%	1.1%	0.2%	0.9%
Lakes and rivers	24.6%	28.6%	11.1%	24.9%	7.4%	11.8%	4.9%	13.5%	4.4%	0.8%	1.0%

A ridge formation runs through the catchment area, and the dominant soil types are mineral soils, mainly moraine, and some highly permeable sand and gravel deposits. There are also some clay soils around Lake Rautajärvi, in the northern part of the Kukkia catchment. Organic soil types (peatlands) cover around 10% of the catchment area, and 75% of these areas are ditched for forestry (Maps of Corine Land Cover and Peatland Drainage Status). Forest ditching was most intensive in the 1960s and 1970s.

Kukkia catchment is located next to the Lammi LTER area. The Lammi LTER area consists of several long-term monitoring and research areas, of which the Evo Forest and Lake Area is the largest and has a special value in terms of long-term ecological studies (see, e.g. ICP Forests, the International Co-operative Programme on Assessment and Monitoring of Air Pollution Effects on Forests).^1^

### Data description

#### Discharge and water quality measurements

The main water quality measurement point in Lake Kukkia was located in the middle of the main basin. Water quality variables, including chemical oxygen demand (COD), were sampled and measured during August–September (Mitikka and Ekholm 2003). The water samples were taken at 5 m layers down from the surface until reaching 25 m. There were 10 sampling days per year on average. In the catchments, measurements of river discharge (daily values) and COD (grab samples; on average, 33 per year) were included in national monitoring programmes (Niemi 2006). In the Kukkia catchment, water quality samples were taken at four stations (sub-catchments 35.783, 35.785, 35.786, 35.787). Discharge measurements were only available in sub-basins 35.786 and 35.785. COD observations were transformed to TOC concentrations by using the formula (TOC = COD × 0.675 + 1.94) presented by Kortelainen (1993) (Figure S1). In Lake Kukkia, the relationship is slightly higher (TOC = COD × 0.753 + 2.108, *R*^2^ = 0.78 and *n* = 90). All this data is available at the open data portal of the Finnish Environment Institute (SYKE).^2^

In the Lammi LTER area, the three catchments had daily observations of discharges and around biweekly observations of DOC concentrations, which forms the main part (around 90%) of TOC loading in Finland (Kortelainen 1993). Water samples were taken at the river outlet by the Lammi Biological Station (LBS) of the University of Helsinki. Sampling started in 1995, but before 2000, the wintertime sampling only took place monthly. In addition, the Evo area also had measurements of soil water and groundwater concentrations of TOC in ICP Forests data.

#### Meteorological and atmospheric deposition data

Meteorological and atmospheric deposition data were obtained from the open data of the Finnish Meteorological Institute. Deposition data was available from the year 1990 onwards. We used the data of the closest measurement stations, which were those at Kotinen (396863, 678762) for deposition data and Lahti Laune (425867, 6759341) for meteorological data (daily observations of precipitation, temperature, humidity, air pressure and wind speed). The data on cloudiness and global radiation originated from Jokioinen Observatory (309562, 6747150).

#### Land-use data

Digital elevation model (DEM) data and other geographical information system (GIS) data were available at an open database provided by the Finnish Environment Institute. Soil data were obtained from Maannostietokanta (Lilja et al. 2006) and land cover data were obtained from the CORINE GIS (for the years 2000, 2006, 2012 and 2018). The CORINE Land Cover dataset provided information on Finnish land cover and land use. The dataset was produced by SYKE, based on an automated interpretation of satellite images and data integration with existing digital map data. Land-use data from before 2000 was derived from the satellite image-based land use and forest classification of Finland (Vuorela 1997) and supplemented with satellite image–based maps of final cuttings in forests on mineral and organic soils, provided by the open data of the Natural Resources Institute Finland. This data is openly available from the year 2009 onwards. Detailed data of agricultural land use was derived from a field parcel database.

### Model description

#### PERSiST

We used a rainfall-runoff modelling toolkit, PERSiST,^3^ with the INCA family of models. PERSiST is designed for simulating present-day hydrology, projecting the possible future effects of climate or land-use change on runoff and catchment water storage. PERSiST has limited data requirements and is calibrated using the observed daily time series of precipitation, air temperature and runoff at one or more points in a river network (Futter et al. 2014).

#### INCA

INCA models (Whitehead et al. 1998) are dynamic mass-balance models, used to track the temporal variations in the hydrological flow paths, nutrient transformations and stores in both the land and in-stream components of a river system. INCA models simulate daily and annual land-use specific nutrient loads for all stores and transformation processes and stores within the land phase, including concentrations in the soil water, ground water and direct runoff. INCA-C is a model for simulating the seasonal and long-term patterns of TOC in soil and surface water (Futter et al. 2007; Lee et al. 2020). Decay of the soil organic C pools is described by first-order kinetics, and the decay rate is limited by temperature and moisture. INCA-C simulates the hydrological fluxes within a catchment or sub-catchments and the masses and fluxes of soil organic carbon (SOC), DOC and dissolved inorganic carbon (DIC) in soils and surface waters (Futter et al. 2007). Within INCA-C, all carbon transformations in soils and surface waters are modelled as a series of first-order differential equations. Atmospheric SO_4_ deposition is used as a surrogate for soil water SO_4_ (Futter et al. 2009).

#### MyLake

Calculations of DOC concentration in the lake were made with the MyLake model. It is a one-dimensional process-based model used for the simulation of the daily vertical distribution of lake water temperature, and thus simulation of the stratification, the evolution of seasonal lake ice and snow cover, and the water chemistry–phytoplankton dynamics (it is described in Saloranta and Andersen 2007).

**Fig. 2:**
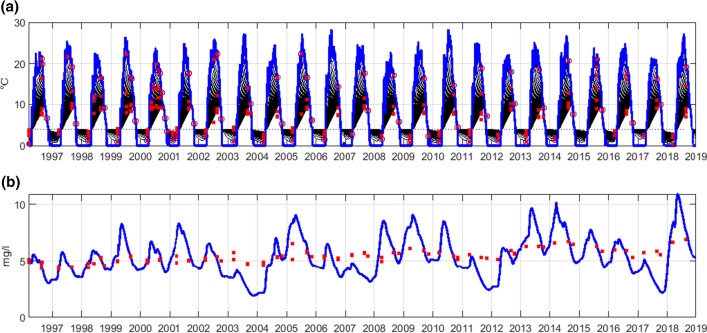
Calibration of the MyLake model (at 0–5 m depth of Lake Kukkia) against observed temperature (**a**) and TOC concentration (**b**). The red square points are observations, and the blue lines are the simulated concentrations at different depths

In the MyLake model, DOC is an input from the surrounding catchment and can be consumed in the lake through both photochemical and biological processes. The bacterial decay of DOC is simulated in a submodule which calculates microbial mineralisation following a three-pool kinetic equation (as described in Vähätalo et al. 2010). Incoming DOC is divided into three pools with different propensities for bacterial mineralisation. The first pool has the fastest decay rate, the second pool has a lower rate and the third pool, which constitutes the largest fraction of DOC, is not affected by bacterial mineralisation.

#### Statistical methods

*Data dredging* is a data mining practice in which large data volumes are analysed in order to look for any possible relationships between the data without forming a hypothesis beforehand. It can be a useful way to find surprising relationships between variables that might not have been discovered otherwise. The weakness is that it may lead to drawing the wrong conclusions, especially if the data have some artefacts. We used Mann–Kendall statistics with Sen’s slope estimation to study monthly increasing or decreasing trends in meteorological and deposition time series. We also used time-series decomposition to remove trends in data with a strong seasonal component.

### Model set-up

Catchment-scale eco-hydrological models (PERSiST, INCA-C) were calibrated against the observed discharge and water quality in the rivers (see Table 2). We used an ungauged catchment approach in simulations. We calibrated the models to LTER catchments and biweekly observations of the DOC concentrations in river water, made since 1995. Parameter values were given in manual calibration to simulate the correct level of process loads and TOC concentration in waters. Manual calibration was finetuned by automatic calibration. We used the goodness-of-fit values of root mean squared error (RMSE) and relative error (RE) to see the general level of simulated concentration against observed concentration. To see how well the simulation fits the observations, we used Nash–Shutcliffe (N–S) efficiency (Aitken 1973) for discharge and a simple *R*^2^ value for concentrations (due to the low number of observations at some stations). The calibration period was 1995–2002 and the validation period was 2004–2013. Automatic calibration, sensitivity and uncertainty analysis was done by using the PEST package (Doherty and Johnston 2003). Litterfall and TOC fluxes from different land-use classes (see Table 3) were calibrated against literature values (Sallantaus 1992; Piirainen 2002; Finér et al. 2003; Nieminen 2004; Mattsson et al. 2005; Kortelainen et al. 2006; Liski et al. 2006; Palviainen et al. 2014; Lehtonen et al. 2016; Nieminen et al. 2017; Manninen et al. 2018). In addition, there were IPC Forest data available with which to calibrate soil processes against observed TOC concentrations in soil water.

**Table 2:** The goodness-of-fit values of the INCA-C model for different sub-catchments

Catchment		Discharge			TOC		
		N–S	RMSE	RE	*R*^2^	RMSE ^2)^	RE
LTER area	35.836	0.649	84.680	− 90.322	0.547	25.910	3.229
LTER area	35.835	0.688	68.113	− 87.551	0.341	34.940	− 20.29
LTER area	35.837	No observations	No observations	No observations	0.100	52.664	− 38.797
Kukkia	35.786	0.501	248.213	− 407.657	0.192	32.216	− 5.713
Kukkia	35.785	0.392	177.898	− 115.993	0.030	73.484	58.796
Kukkia	35.787	No observations	No observations	No observations	0.114	40.314	− 4.583
Kukkia	35.783	No observations	No observations	No observations	0.133	65.732	58.032

*N–S* Nash–Shutcliffe efficiency, *RMSE* root mean square efficiency, *RE* relative error

**Table 3:** Process loads in different land-use classes compared with values reported in literature

	TOC export (kgC ha^−1^ a^−1^)			Litterfall (kg ha^−1^ day^−1^)	
	Simulated	Literature values		Input	Literature values
		Mean	Variation		Mean
Forest on mineral soil	21.33	34^a^	6–133	3.3	2.6–5.5^g^
Forest on organic soil	58.50	71^b^	6–133	10	7.9–11.8^h^
A forest cutting on mineral soil	29.77	39^c^	6–143	4.8	1.2–4.8^i^
A forest cutting on organic soil	72.53	91^d^	18–175	7.9	3.3–5.5^j^
Agriculture	45.57	45^e^	25–65	7	3–15^k^
Open pristine mires	90.83	106.5^f^	93–120	5	–

^a^Mattsson et al. (2005), Kortelainen et al. (2006), Palviainen et al. (2014) and Nieminen (2004)

^b^Mattsson et al. (2005), Kortelainen et al. (2006), Palviainen et al. (2014) and Nieminen et al. (2017)

^c^Nieminen (2004) and Palviainen et al. (2014)

^d^Palviainen et al*.* (2014) and Nieminen et al. (2017)

^e^Manninen et al. (2018)

^f^Sallantaus (1992)

^g^Piirainen (2002), Finér et al. (2003), Liski et al. (2006) and Lehtonen et al. (2016)

^h^Piirainen (2002), Finér et al. (2003), Liski et al. (2006) and Lehtonen et al. (2016)

^i^Piirainen (2002) and Liski et al. (2006)

^j^Piirainen (2002) and Liski et al. (2006)

^k^Yli-Halla, M

This calibration of terrestrial area was then transferred to the Lake Kukkia sub-catchments. These sub-catchments do not have observations of TOC/DOC concentrations; they only have observations of COD concentration. Thus, calibration was checked against the observations of COD, which are known to correlate with DOC/TOC concentration (Kortelainen 1993; Rantakari et al. 2004). The lake chains in the Kukkia catchment were calibrated as part of the river network by using the physical properties and retention time. The general level of simulated TOC concentration and that calculated from COD observations were at the same level (they had the same RMSE and RM values) though the dynamics were at a lower level than in the LTER sub-basins.

The MyLake model was calibrated against observed temperature and TOC (calculated from COD) concentration in the lake (Fig. 2).

### Scenarios of environmental change

We analysed the long-term time series of historical observed data (ranging from 1995 to 2019) of atmospheric SO_4_ deposition, air temperature and precipitation to look for possible trends. The trends found were filtered out using time-series analysis and filtered time series were used as scenarios for situations without change. We simulated static situations without an increasing trend in air temperature (*no climate change*) and without a decreasing trend in atmospheric deposition (*no deposition change*). For land use, change in the years when the forest cutting area was lowest and highest were used as the scenario (*no forest cutting*). Altogether we had to simulate the following four options:

1. A baseline (base)
2. Forest cuttings at the same level as in the 1990s, but current climate and current SO_4_-S deposition are used (Scenario A)
3. No increasing trend in air temperature, but current precipitation, SO_4_-S deposition and area of forest cuttings are used (Scenario B)
4. No decreasing trend in SO_4_-S deposition, but the current climate and area of forest cuttings are used (Scenario C)

## Results

### Observed trends in water quality

**Fig. 3:**
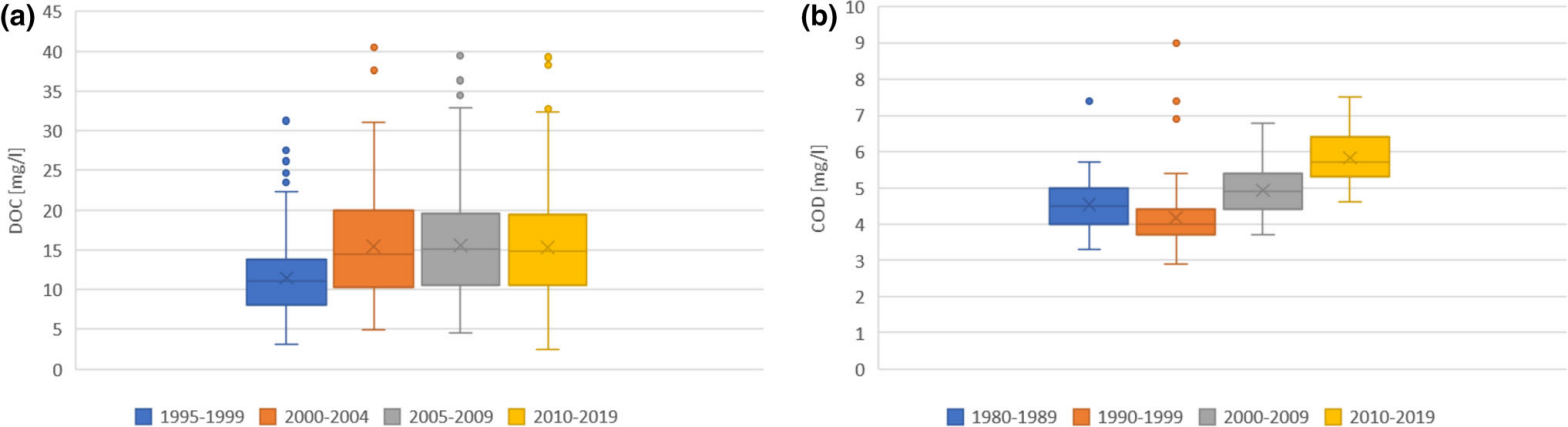
The observed trends in **a** DOC concentration in the LTER-area river, Mustajoki, **b** COD concentration in Lake Kukkia

There has been a clear brownification process for the surface waters in the area. In the LTER areas, the river-water concentration of DOC increased between periods 1995–1999 and 2000–2004 (*p* < 0.01), but since 2004, there were no statistically significant increases (see Fig. 3). There was a significant increasing trend in COD concentration in Lake Kukkia since 1990 (*p* < 0.001), but no clear change between periods 1980–1989 and 1990–1999 (see Fig. 3).

In the dredge analysis, the water quality of Kukkia was best explained by precipitation, the number of rainy days and the forest cut area. In addition, atmospheric deposition plays role in explaining the COD concentration.

### Observed trends in deposition, climate and runoff

Atmospheric deposition has clearly decreased during recent decades in Finland (Vuorenmaa et al. 2018). At the Kotinen station, the SO_4_ deposition was highest in the early 1990s at up to 460 mg m^−2^, and now it has levelled off to be around 100 mg m^−2^. The decrease has been statistically very significant (*p* < 0.001). (Figure S2).

Annual mean precipitation varied between 470 and 815 mm at the Lahti Laune station during 1985–2019, but there was no significant increasing trend. The only trend (*p* < 0.05) was observed in March, when precipitation decreased. The number of rainy days in a year varied between 159 and 216. This number showed a decreasing trend (*p* < 0.05).

The annual mean air temperature was 4.7 °C, and it varied between 1.9 and 6.1 °C. It showed a statistically significant (*p* < 0.05) increasing trend. The temperature increased, especially in early autumn (September) and late autumn or early winter (November–December) (see Table 4).

**Table 4:** Trends in air temperature, precipitation and runoff (Mann–Kendall analysis)

Month	Air temperature			Precipitation			Runoff		
	Significance	Sen’s slope	Constant	Significance	Sen’s Slope	Constant	Significance	Sen’s slope	Constant
January		− 0.032	− 4.39		− 0.142	50.53		0.077	8.03
February		0.088	− 9.87		− 0.190	36.49		− 0.016	6.35
March		0.007	− 2.11	*	− 0.571	46.80		− 0.004	9.07
April		0.059	1.17		0.300	21.80		− 0.380	63.06
May	+	0.053	7.86		− 0.355	46.05	***	− 1.120	54.18
June		− 0.009	14.45		0.800	43.50	*	− 0.294	17.87
July		0.044	15.00		− 0.853	88.94	+	− 0.137	9.37
August	*	0.045	13.41		− 0.557	92.96	*	− 0.180	8.56
September	**	0.065	7.41		− 0.715	74.04	*	− 0.196	10.91
October		− 0.008	4.90		− 0.075	58.78	*	− 0.492	27.47
November	*	0.110	− 4.72		− 0.050	61.025		− 0.177	22.80
December	*	0.166	− 9.81		0.215	42.55		0.352	12.36
Mean	**	0.043	3.14	+	− 2.726	670.53	**	− 3.567	296.76

+< 0.1, *< 0.05, **< 0.01, ***< 0.001

Annual runoff showed a statistically significant decreasing trend. The runoff especially decreased in early summer (May–June) and autumn (August–October). (Figure S2).

### Land-use/-cover change

**Fig. 4:**
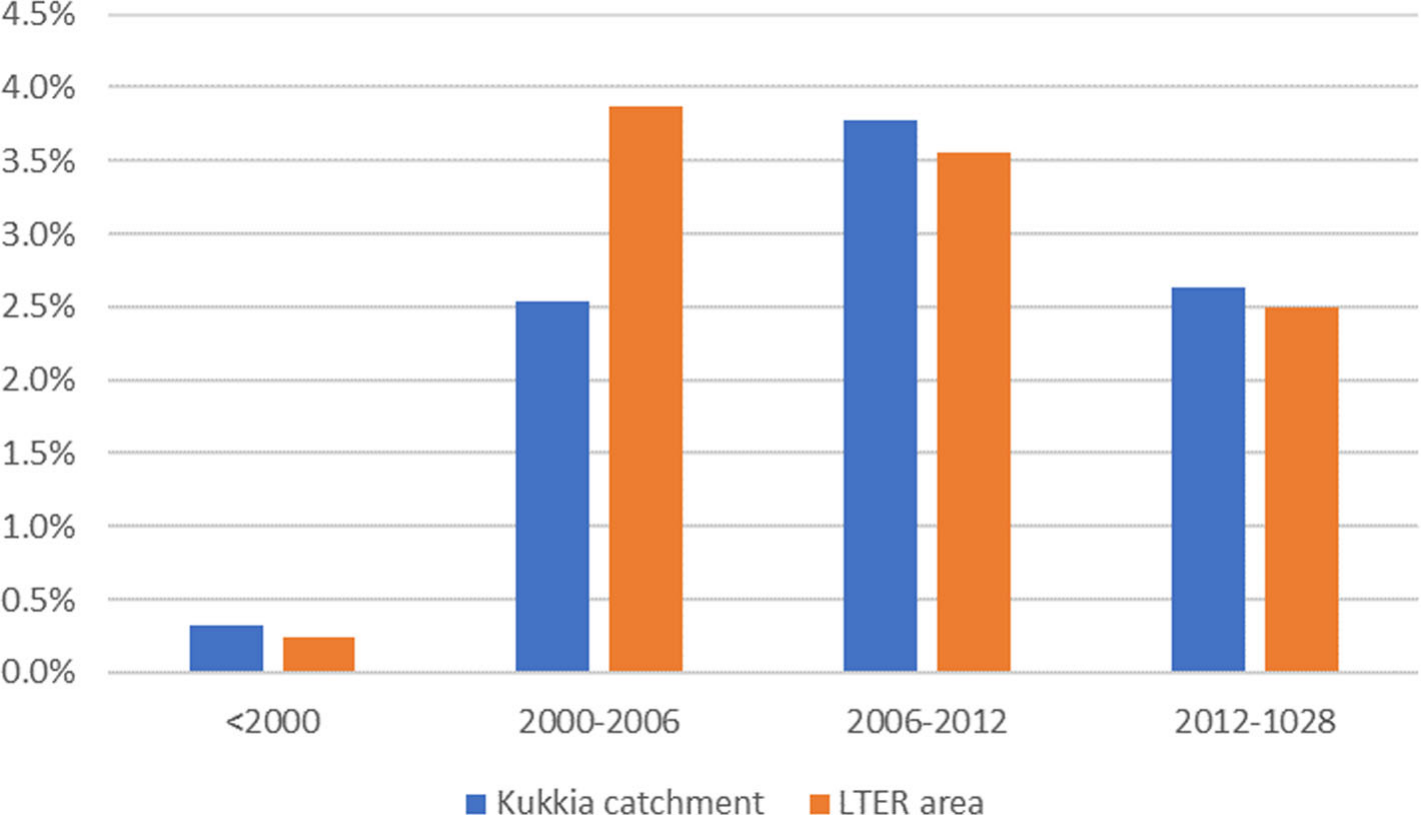
Changes in the forest-cut area in different periods, shown as a percentage of the total area

The major land-use change has occurred in economically used coniferous forests. The area of forest cutting has increased since 1985 (see Fig. 4), and the area was largest between 2006 and 2012 in the Kukkia catchment. In the LTER area, the forest cutting area was the largest between 2000–2006. Logging areas are determined from satellite images as land-use change in a cell.

The second largest land-use change was the afforestation of open areas, which has accounted for around 1–2% of the total catchment area since 1985. There were no significant changes between other land-use forms. In the early 1990s, the area of forest cuttings was smaller than the area of forests changing from open areas/forest cut areas to growing forests. Since 2000, it was the contrary and it has stayed like that throughout the 2000s.

Since 2009, site-specific data of dominant tree species has been available at the Natural Resources Institute Finland as open data. There is a large variation in spruce fir biomass, but no increasing or decreasing trend can be observed (Figure S3). In a summary of the development of Finnish forests, spruce’s proportion of the productive forest area is decreasing at landscape level in Southern Finland (Korhonen et al. 2021).

### Modelled trends in water quantity

In the discharge pattern, there was a change between Scenario B (*No trend in air T*) and other scenarios so that annual discharge is about 10% lower than that of the current climate (1985–2019). The spring discharge peak was lower and occurred earlier. In addition, winter runoff was higher. This change mainly reflected the change in the timing of the snow melt. (Figure S4).

Simulated actual evapotranspiration was almost 5% higher in the current climate than it was without an increasing trend in air temperature (1985–2019), but there was no shift in the timing of peak evaporation. Thus, changes in evaporation explained the difference in summer discharge. Forest cutting only had a minor influence on discharge (Figure not shown).

### Modelled trends in terrestrial ecosystems

**Fig. 5:**
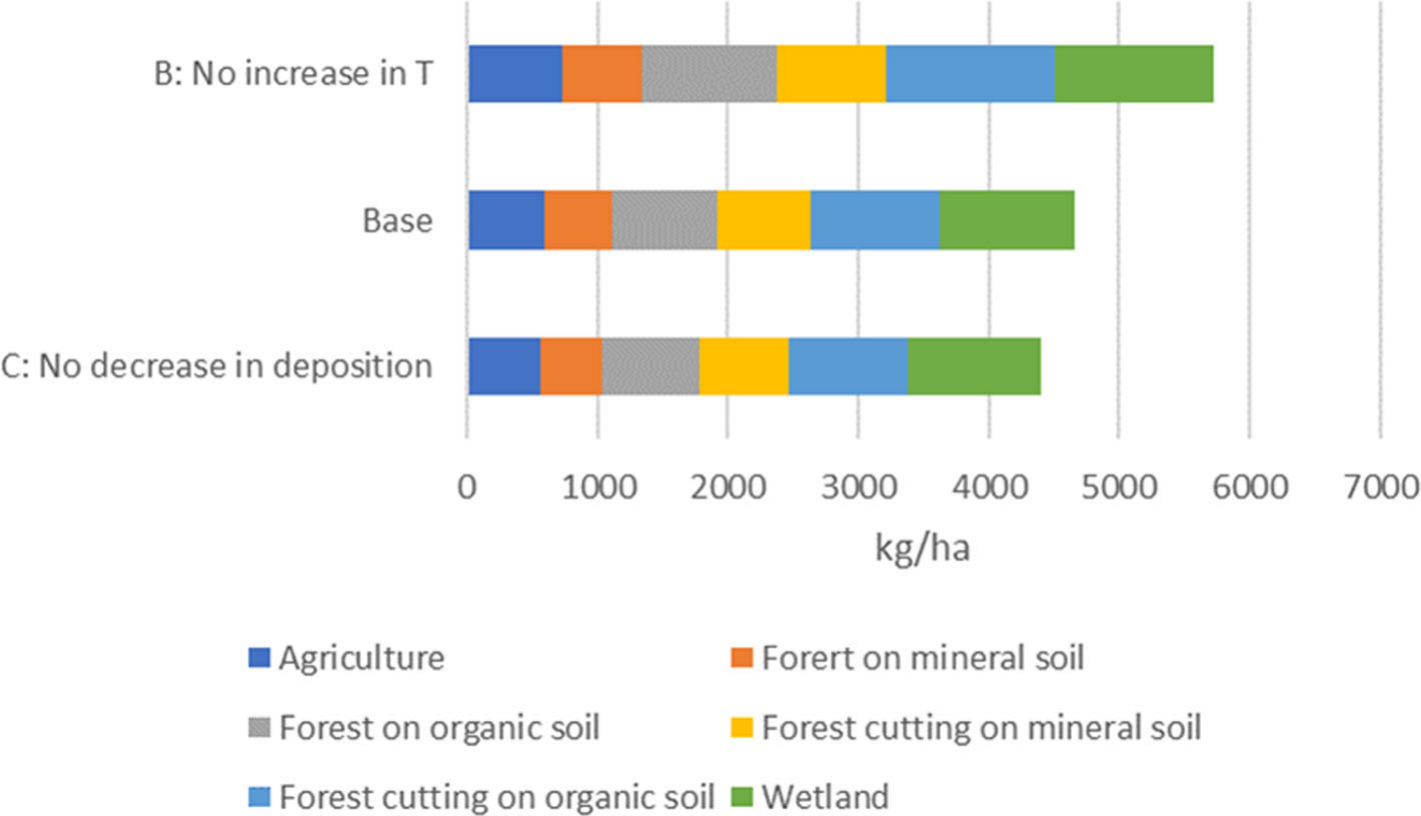
TOC output from different land uses, based on the model results

There was a clear decreasing trend in SO_4_ deposition during 1995–2019, which resulted in the slightly higher leaching of TOC from terrestrial ecosystems. However, in the Kukkia catchment the effect was small, about a 5% increase in the terrestrial TOC load. In addition, the smaller runoff in the current climate decreased the terrestrial TOC leaching. Without an increasing trend in temperature, the terrestrial TOC load was about 25% higher than the current load. These environmental changes have influenced all the land-use classes almost equally. Of the individual forms of land use, forests had the greatest impact on TOC leaching. In the sensitivity analysis, the INCA model proved to be the most sensitive to changes in litter input. Forest cut areas formed almost 40% of the TOC load, especially for those forests on organic soils (see Fig. 5).

### Modelled changes in lake water quality

**Fig. 6:**
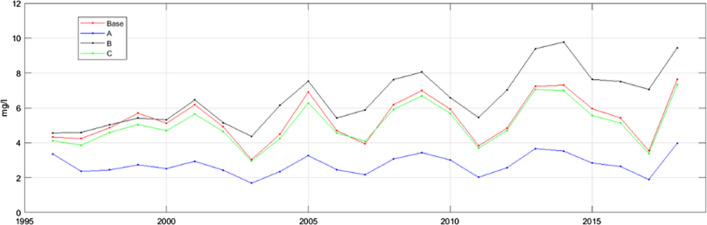
(Scenario A) Forest cutting at the same level as in the early 1990s; (Scenario B) no increasing trend in air temperature; (Scenario C) deposition at the same level as in the early 1990s

Forest cuts increased the TOC concentration in the lake (see Fig. 6). This is seen in Scenario A, which is based on lower cutting rates than the baseline. An increased temperature decreased the DOC concentration in the lake by decreasing runoff and leaching from the catchment due to higher evapotranspiration. The influence of atmospheric deposition on lake-water DOC concentration was low.

On the lake level, the dynamics of DOC concentrations were simulated relatively well, but concentrations in some extremely dry periods (2003–2004, 2007–2008 and 2017–2018) were underestimated. This could be due to the fact that the depth of Lake Kukkia and the lake chain above it equalised the concentration fluctuations when the terrestrial input was low.

The simulated base scenario showed an increasing trend in annual mean concentrations (*p* < 0.1), which disappeared in Scenario A (*No forest cuts*). Instead, in Scenario B (*No increase in air temperature*) the increasing trend became statistically very significant (*p* < 0.001).

## Discussion

Climate change is assumed to increase both air temperature and precipitation in Finland (Ruosteenoja and Jylhä 2021). The observed mean temperature has already very likely to have risen over 2°C during the years 1847–2013, which amounts to a 0.14°C rise per decade (Mikkonen et al. 2015). This is consistent with human-induced global warming. In the Lake Kukkia catchment, the annual mean temperature has increased by 2°C since 1985. At the same time, we did not find any increase in annual precipitation. This is in line with the observation of Hohenthal (2009) that precipitation has decreased rather than increased in southern Finland. The spring discharge peak occurs earlier and is lower than in the scenario without climate change. The changes in the peak flow reflect the change in snow melting and summer low-flow period changes in evaporation. Due to the smaller runoff, the TOC leaching from terrestrial areas decreased. This finding is in line with the recent results of Sharma et al. (2023) regarding the importance of the hydroclimatic regime on terrestrial TOC leaching.

There was clear decreasing trend in SO_4_ deposition. In general, the level of atmospheric SO_4_ deposition has been low in Finland (Vuorenmaa et al. 2017). The decrease in acidic deposition has clearly resulted in a higher leaching of TOC according to theory (e.g. the theories of de Wit et al. 2007 and Monteith et al. 2007). However, in the Kukkia catchment area, the effect is small. In the previous statistical analysis of Finnish rivers, SO_4_-S deposition was one factor (but not the most important factor) explaining changes in TOC concentration (Cano Bernal et al. 2022).

Forest cut areas formed a considerable share of the terrestrial TOC load. The volume of forest cuttings has shown an increasing trend since 1985, though in the early 1990s, the volume temporarily decreased (LUKE statistics). In particular, forest cuttings on organic soils have a great impact, even when considering that the share of such organic soil types in the catchment is small. The forest cuttings on the organic soils are typically combined with forest ditching, which has been observed to influence water quality more than forest cutting alone. Forestry measures are assumed to influence water quality for about 10 years (Finér et al. 2020, 2021), although recent studies have shown that the influence time of forest ditching may be much longer (Nieminen et al. 2017, 2018, 2020, 2021). This is in line with the observation by Cano Bernal et al. (2022) that the area of forest ditching is the largest single factor that explains the TOC concentration in the rivers and TOC loading from catchments. According to the preliminary information of 2021, there is a difference between tree growth and removal due to increased felling and reduced growth (Haakana et al. 2022). This change in land use is seen in the Kukkia catchment as well. In the early 1990s the area of forest cuttings was smaller than the area of afforestation (the change from open areas/forest cut areas back to forests). Since 2000 it has been the contrary. This study shows that changes in forest cuts have a significant effect on water TOC concentration. The area of spruce coverage may later affect the browning of the waters when the later forestry practices promote the formation of highly spruce dominated stands (Škerlep 2021; Williamson et al. 2021).

## Conclusions

This study shows that changes in forest cuts have a significant effect on water quality. The changes have formed the primary reason for the brownification of Lake Kukkia. The decrease in acidic deposition has resulted in a higher leaching of TOC, but in the Kukkia catchment area, the effect is very small. In the Lake Kukkia catchment, the annual mean temperature has increased by 2 °C since 1985. At the same time, we did not find any increase in annual precipitation. Due to the smaller runoff, the TOC leaching from terrestrial areas is smaller than it would have been without the increasing trend in temperature.

### Supplementary Information

Below is the link to the electronic supplementary material.

Supplementary material 1 (PDF 616 kb)

## Footnotes

1 http://icp-forests.net/.

2 https://www.syke.fi/opendata.

3 https://www.persistmodel.org/home/landing.
