# Supplementary material for: Quantification of the effect of environmental changes on the brownification of Lake Kukkia in southern Finland
Source: Ambio. 2023 Sep 21;52(11):1834–46. doi: 10.1007/s13280-023-01911-7 (PMC10562317; doi:10.1007/s13280-023-01911-7)
Supplement: Supplementary file 1 — Supplementary material 1 (PDF 616 kb) [file 13280_2023_1911_MOESM1_ESM.pdf]

Quantification of the effect of environmental changes on the brownification of Lake Kukkia in southern Finland

Katri Rankinen[1*], Virpi Junttila[1], Martyn Futter[2], José Enrique Cano Bernal[1], Daniel Butterfield[3], Maria Holmberg[1]

1. Finnish Environment Institute

2. Swedish University of Agricultural Sciences

* Correspondin author

e-mail: katri.rankinen@syke.fi

Authors do not have any conflict of interest

Quantification of the effect of environmental changes on the brownification of Lake Kukkia. Ambio. Rankinen et al.

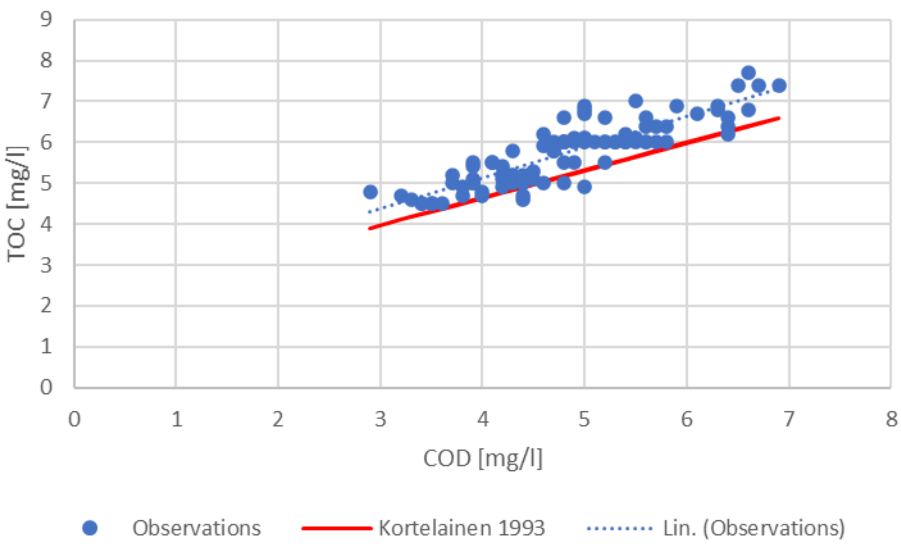

Figure S1. Relasionship between COD and TOC observations in the lake Kukkia (n=90)

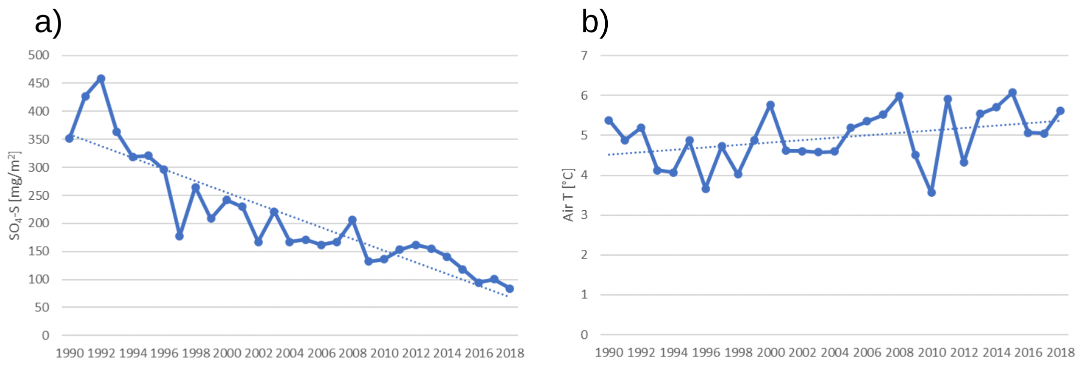

Figure S2. Observed trends in (a) SO_4-S deposition and (b) annual mean air temperature

a)

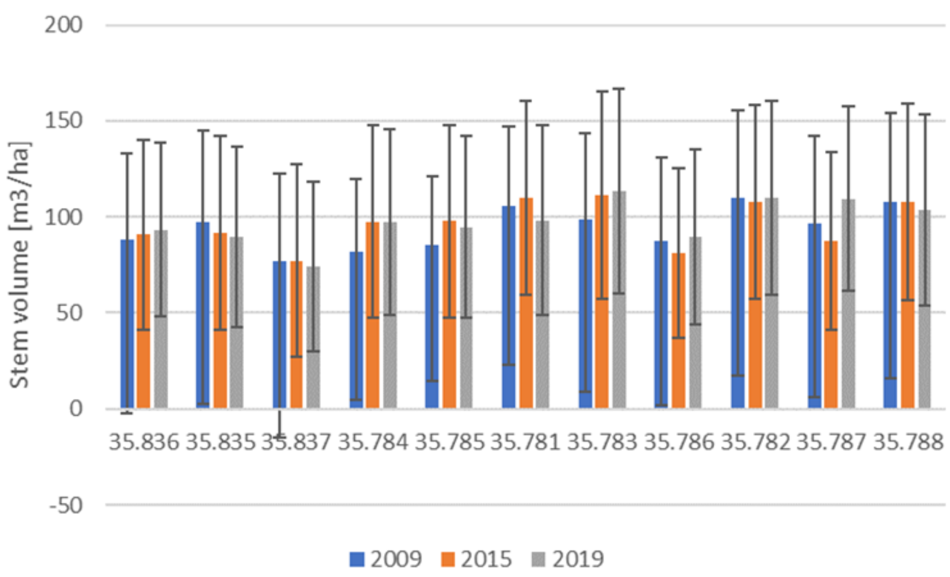

b)

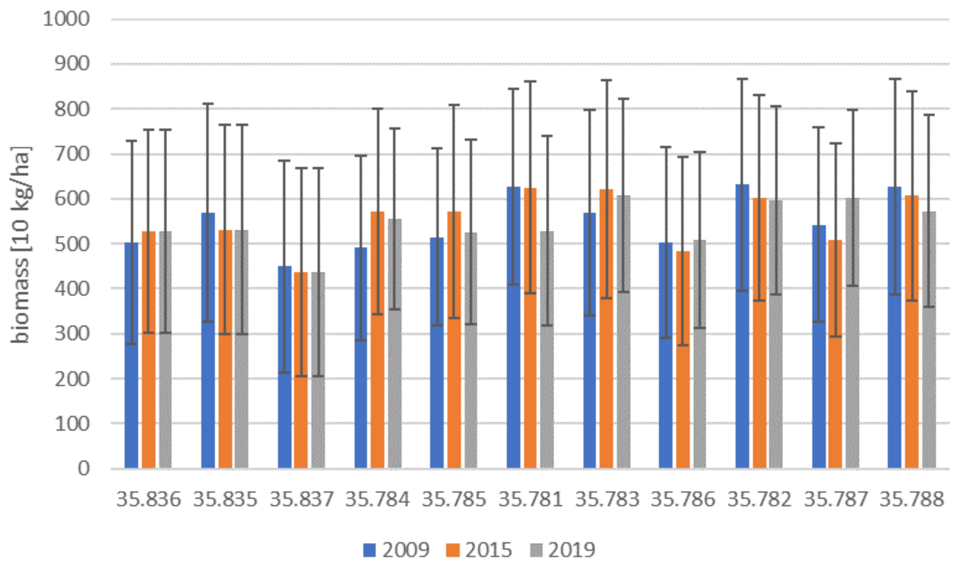

Figure S3. Spruce a) needle biomass; and b) stem volume in different sub basins

a) b)

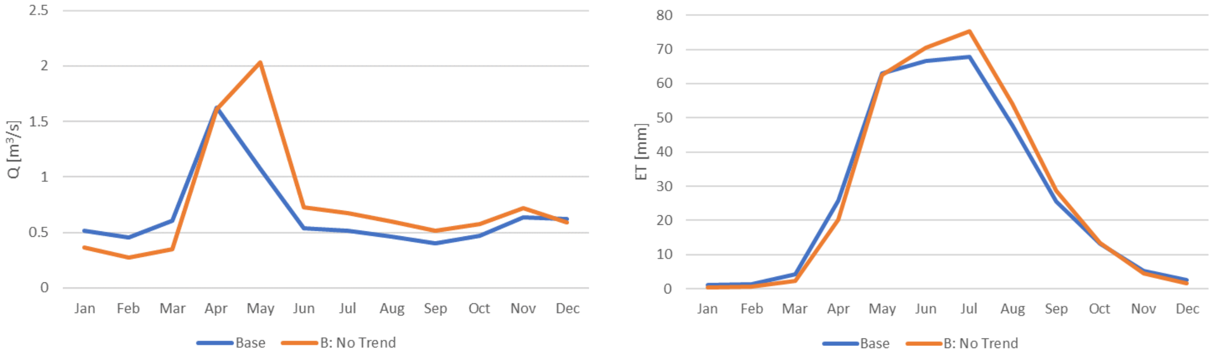

Figure S4. Timing of (a) peak discharge and (b) evapotranspiration at the Mustajoki catchment
